# Advanced Hydrogels as Exosome Delivery Systems for Osteogenic Differentiation of MSCs: Application in Bone Regeneration

**DOI:** 10.3390/ijms22126203

**Published:** 2021-06-08

**Authors:** Elham Pishavar, Hongrong Luo, Mahshid Naserifar, Maryam Hashemi, Shirin Toosi, Anthony Atala, Seeram Ramakrishna, Javad Behravan

**Affiliations:** 1 Biotechnology Research Center, Pharmaceutical Sciences Research Institute, Mashhad University of Medical Sciences, Mashhad 91735, Iran; elpishavar@gmail.com (E.P.); mahshidnaserifar@yahoo.com (M.N.); hashemim@mums.ac.ir (M.H.); Tusi1061@gmail.com (S.T.); 2 Wake Forest Institute for Regenerative Medicine, Wake Forest School of Medicine, Medical Center Boulevard, Winston-Salem, NC 27157, USA; aatala@wakehealth.edu; 3 Engineering Research Center in Biomaterials, Sichuan University, Chengdu 610064, China; hluo@scu.edu.cn; 4 Center for Nanofibers and Nanotechnology, Department of Mechanical Engineering, National University of Singapore, Singapore 117581, Singapore; 5 School of Pharmacy, University of Waterloo, Waterloo, ON N2G 1C5, Canada; 6 Center for Bioengineering and Biotechnology, University of Waterloo, Waterloo, ON N2G 1C5, Canada

**Keywords:** bone tissue engineering, exosome, advanced hydrogels

## Abstract

Hydrogels are known as water-swollen networks formed from naturally derived or synthetic polymers. They have a high potential for medical applications and play a crucial role in tissue repair and remodeling. MSC-derived exosomes are considered to be new entities for cell-free treatment in different human diseases. Recent progress in cell-free bone tissue engineering via combining exosomes obtained from human mesenchymal stem cells (MSCs) with hydrogel scaffolds has resulted in improvement of the methodologies in bone tissue engineering. Our research has been actively focused on application of biotechnological methods for improving osteogenesis and bone healing. The following text presents a concise review of the methodologies of fabrication and preparation of hydrogels that includes the exosome loading properties of hydrogels for bone regenerative applications.

## 1. Introduction

In bone-related diseases, including bone defects, fractures, and tumors, and periodontitis, the regeneration of the lost bone is a critical consideration [1]. Bone resorption and formation happen through osteoblastic and osteoclastic activity.

Bone responds to injury by consecutive reactions, including inflammation, activation of the repair mechanisms, and various tissue remodeling phases [1,2]. Each of those phases is distinct by the cellular and molecular factors involved and the stage-specific tissue status, although they partially overlap in time [3].

The inflammatory phase, which includes bleeding from the fracture and damage to the surrounding soft tissue, leads to the release of inflammatory cytokines. Subsequently, mesenchymal progenitor cells proliferate at the site of injury and differentiate into osteoblasts and chondrocytes [4,5]. In addition, there are important factors that affect the procedure of mesenchymal stem cells’ differentiation into chondrocytes or osteoblasts. These factors, among many others, include hypoxia, bone morphogenetic proteins (BMPs), and nanoparticles. The initial hypoxic condition results in the exploitation of the pro-angiogenic factors vascular endothelial growth factor (VEGF), angiopoetin-1, and platelet-derived growth factor (PDGF) and consequently leads to new blood capillary formation. These capillaries then grow and advance to the injury site [6,7]. PDGF is an important regulator of fracture healing. It acts by stimulating both osteogenic and angiogenic pathways [5,8].

The use of MSCs is the preferred method in the delivery of recombinant growth factors. However, due to the limitations of manufacturing and challenges in the application of MSCs in the clinical setting, new approaches to bone tissue engineering are heavily sought after [9]. Furthermore, the secretome of MSCs provides a cell-free alternative containing a cocktail of various proteins and peptides, including growth factors and cytokines with paracrine properties, harboring angiogenic potential and anti-inflammatory effects proven to enhance bone regeneration in vivo [10].

Exosomes are released by various cells, including MSCs. They are considered to be efficient means of communication between the cells for growth and functional purposes. Exosomes are also actively involved in tissue damage repair. Internalization of exosomes by other cells facilitates the exchange of mRNAs, peptides, and proteins, thereby affecting target cells involved in tissue repair. Exosomes, if unformulated, are rapidly cleared from the body via fluids. However, formulations of exosomes in a protective hydrogel may protect them from degradation and provide a sustained reservoir for therapeutic effects [11].

It has been shown that manipulating the physicochemical properties of the used biomaterial surface can improve the healing of injured organs or damaged tissues. It is suggested that the physicochemical and mechanical properties of the biomaterial surface could support the cells’ survival and stimulate neo-tissue formation [12]. To provide biomaterials with optimal physicochemical properties matching those of the target tissue, various methods for fabrication and template construction, including 3D printing, self-assembly, electrospinning, and phase-separation, are used [13].

## 2. Biomaterials in Bone Regeneration

Currently, for the treatment of bone loss conditions (replacement of missing or diseased bone), the gold standard procedure is bone grafting. The bone graft may be autologous, heterologous, or synthetic [14]. Most studies in this field are focused on less invasive, less expensive, safer, and easier-to-apply methodologies using synthetic bone grafts that contain biomaterials such as tricalcium phosphate (TCP), hydroxyapatite (HA), bioactive glass, and ceramics [15,16]. These biomaterials provide osteo-conduction but have little osteo-induction activity. Therefore, combination of these biomaterials with osteo-inducing materials, including growth factors, hormones, and drugs, can enhance their application potential [14]. Intensive studies are now underway to explore the application of different types of biomaterials (polymeric, ceramic, metallic, or composite) combined with biologically active compounds (e.g., phytohormones, hormones, platelet lysates, and growth factors) or various types of drugs (e.g., selective estrogen receptor modulators such as raloxifene, lipid-lowering agents such as simvastatin, and bisphosphonates such as alendronate) for bone tissue conditions [17,18].

Based on their origins, biomaterials for bone regeneration are classified into the following three different sub-groups: (1) biomaterials of natural origin, consisting of harvested autologous bone grafts as well as allogenic grafts, including demineralized bone matrix, natural bone hydroxyapatite from animal bones, and naturally occurring non-animal materials (bone-analogue calcium phosphate from marine algae); (2) synthetic (alloplastic) materials consisting of ceramics such as TCP, bioactive glasses, and hyaluronic acid (HA); and (3) composite materials combined of different materials, including polymers and ceramics [19]. Ideally, a composition sponge for bone substitution would also consist of several biofactors, including an osteo-conductive structure, osteogenic cells, and osteo-inductive factors, to provide sufficient mechanical, physical, and chemical properties and to promote proper tissue vascularization [2,19].

The essential properties of a bone engineering scaffold include biodegradability, biocompatibility, mechanical strength, and a porous structure to support osteogenic cells’ attachment and penetration and to promote vascularization. Moreover, it should be able to reduce the chance of complications, including post-operation stress shielding [15]. While a wide range of pore sizes for bone regeneration studies have been used, the minimum pore size of scaffolds for considerable bone tissue growth is 75–100 µm with an optimal pore size of 100–135 µm. This promotes significant bone regeneration and supports angiogenesis [20,21]. Scaffolds may also deliver materials, such as growth factors and antibiotics, to support the cells and growing bone tissue [14,22].

For bone tissue regeneration, hydrogels can be considered to be highly attractive scaffolds and exceptionally promising alternative materials. This is due to their suitable properties that include their excellent elasticity, biocompatibility, biodegradability, and mechanical properties [23].

### 2.1. Hydrogel Scaffolds for Bone Tissue Regeneration

Hydrogels are three-dimensional systems with high water content and hydrophilic polymer chains harboring special traits, including biocompatibility and elasticity, with the possibility of modification of their chemical properties. They are also able to mimic the extracellular matrix (ECM) and are capable of acting as a cell/tissue growth medium. These properties of hydrogels justify their broad use in biomedical research, from drug delivery to a vast range of applications in regenerative medicine. Their ability to encapsulate cells and increase the proliferation and retention of the cells is also of critical importance [24,25].

#### 2.1.1. Natural Hydrogels

Natural materials, such as polysaccharides (chitosan, hyaluronan, and alginate) and proteins (fibrin, fibroin, collagen, and gelatin), can be used for the preparation of hydrogels [26]. Desirable properties of natural hydrogels for application in bone repair include biodegradability and proper cell interactions. The downside of natural materials for hydrogel preparation is their low stability, undesirable mechanical properties, and lower levels of cell adhesion. Specific peptide sequences, such as the arginine–glycine–aspartic acid (RGD) sequence, are used to improve the cell adhesion property of an alginate. Moreover, porosity and stiffness are important factors that affect the fate of cells in the developing tissue [26]. Several different natural hydrogels have been studied as scaffolds for bone regeneration (Table 1).

Hyaluronic acid (HA) is a polysaccharide with non-immunogenic properties. It is a major contributor to the ECM’s structure and has a very important role in tissue regeneration, angiogenesis, wound repair, molecular and cellular signaling, organization of the matrix structure, and skin tissue morphogenesis [26]. Chitosan is another natural polymer with a linear structure. It has a structure composed of β-(1,4)-linked D-glucosamine and N-acetyl-D-glucosamine units [27]. This natural polymer possesses intrinsic wound healing properties.

Collagen is the major protein component of ECM in mammalian tissue. Chemical and physical methods may be used to cross-link collagen molecules. Collagen type I can enhance interaction between the gel surface and cells through covalent cross-linking [28,29]. Gelatin is produced by partial and irreversible hydrolysis of collagen. Gelatin contains many RGD peptide sequences that enhance cell attachment. This sequence is also able to target specific amino acid sequences of matrix metalloproteinase (MMP) to promote cell remodeling processes. In comparison with collagen, gelatin is less immunogenic and exhibits higher water solubility [30]. A gelatin derivative, gelatin methacryloyl (GelMA), is considered a suitable biodegradable polymer. However, its use in bone regeneration has some disadvantages, including low mechanical strength, a high swelling rate, and a short degradation time. Nevertheless, the incorporation and amplification of various cells in GelMA hydrogels have been used extensively due to their combined properties of mechanical characteristics, the presence of bioactive peptide sequences, and biocompatibility [31]. It has been shown that a combination of poly ethylene glycol diacrylate and GelMA (PEGDA/GelMA) creates a hydrogel with stronger mechanical properties and appropriate degradation rates compared with pure GelMA hydrogel [32]. Moreover, the incorporation and proliferation of various cell types in GelMA hydrogels have been extensively used. This is due to the presence of bioactive peptide sequences, the combined biocompatibility, and mechanical properties [33].

Creation of 3D structures similar to natural tissues, consisting of all the components involved in the makeup of dynamic tissue (living and dividing cells and matrix biomaterials) in three dimensions is an exciting prospect of tissue engineering. Decellularization of a tissue of interest by removing the cells can preserve the ECM and the exact 3D aspects of the tissue. Therefore, this approach might be considered to be one of the best choices [34]. For a bone ECM production process, decellularization and demineralization of bone matrix are needed. Smith et al. produced an ex vivo model for bone regeneration by mixing an alginate with a decellularized and demineralized bovine-derived bone matrix [35]. A gelatin–chitosan nanocomposite containing hydroxyapatite and titanium dioxide nanoparticles has shown biocompatibility, good biodegradability, and suitable bone induction. This can be used as a promising substitute for a bone regeneration membrane with great potential in orthopedic applications [36].

#### 2.1.2. Synthetic Hydrogels

For bone repair and regeneration, hydrogels may be synthesized from a range of polymeric biodegradable materials, such as polyethylene glycol (PEG), Sanya methyl carbonate and the relevant copolymers, polyvinyl alcohol (PVA), poly lactic acid, and polyacrylamide (PAM). Synthetic polymers, as compared to natural materials, possess the basic structural units, defined properties, including porosity and degradation time, and known mechanical properties [37]. These properties make them suitable for specific bone tissue regeneration applications. There are several advantages to using hydrogels made from synthetic polymers. Advantages include their reliable material sources, longer shelf life, and lower risk of immunogenicity. Moreover, they can be used as suitable delivery means for active tissue growth factors, proteins, and medicinal supplements to the growing bone tissue. Lee and coworkers used novel degradable and injectable hydrogels consisting of adipic acid dihydrazide and poly aldehyde guluronate (PAG) to deliver primary rat cranial osteoblasts trough subcutaneous injection into the back of mice [38]. They reported that mineralized bone in the mouse’s backbone was formed nine weeks post injection [39]. Thoma and co-workers prepared a few polyethylene glycol (PEG) hydrogels and classified them into six groups based on the density of the gels (physical modification). The PEG hydrogels were structurally and functionally modified with the incorporation of RGD by chemical modification. Each hydrogel construct was implanted into six different loci of rabbit skulls. Following a six week observation, the investigators found that the particular type of physical or chemical modification had a significant influence on the stability of the PEG hydrogel matrix, the degradation time value, and the integration of the hydrogel into the surrounding soft tissues and hard tissue [40]. Various chemical reactions, including click chemistry, free radical polymerization, and thiol-ene chemistry, may result in the formation of covalent cross-linked hydrogels [41,42].

## 3. Preparation of Hydrogel Scaffolds

Based on the intended application, hydrogels can be synthetized with different shapes and sizes. This may include, for example, the preparation of microgels and nanogels by microfluidics. Moreover, the concentration of the polymer or processing parameters can affect the size of the hydrogel particles [39]. Repeatable scaffolds with a controlled hierarchal porous structure can be obtained by the optimal fabrication technique, which impacts on both the mechanical and biological response of bone tissue [37]. Current methods for producing bone tissue scaffolds are discussed below.

### 3.1. Hydrogel Fibers

Hydrogel fibers consist of a fibrous texture with variable sizes from several nanometers to several microns. Hydrogel fibers are used in tissue engineering due to their high surface-to-volume ratio, rapid cell interaction, and immobility. Hydrogel fibers ensure cell viability and cell dispersion. The use of hydrogel fibers in bone regeneration has shown great potential in tissue engineering. Preparation of hydrogel fibers usually consists of two steps: a spinning process and a linking process. Generally, various types of spinning methods are used: electric spinning, microfluidic spinning, wet spinning, gel spinning, 3D printing technology, and hydrodynamic spinning, among which electric spinning and microfluidic spinning are the most common methodologies. Gelatin is obtained from the degradation of collagen and is an excellent polymer for the production of hydrogel fibers. This is due to its natural binding to the arginine–glycine–aspartic acid peptide (RGD). Compared with microbeads, hydrogel fibers can be injected at the defect site with a syringe and remain at the implant site for longer periods of time. Perez et al. co-delivered cobalt (Co) and BMP with well-tunable core shell hydrogel fiber scaffolds to induce angiogenesis and ossification in a rat calvarial defect [43]. On the other hand, hydrogel fibers have disadvantages such as poor mechanical strength and high swelling properties.

Poly vinyl alcohol (PVA) hydrogel fibers are highly swollen and therefore burst and release their components. To reduce the unwanted adverse effects due to the sudden release of the incorporated medication and growth factors, a research team successfully extended the duration of the drug by modifying the surface of the hydrogel fibers. Im et al. found that carbon–fluorine (c-f) bonding to the surface of PVA electrospun fibers through fluorination could significantly reduce the probability of a fiber reactive dye (Procion Blue) bursting and increased the diffusion time by 6.7-fold [44]. Furthermore, due to the stretched structure of hydrogel fibers, they lack favorable mechanical properties. Composite materials have been prepared from hydrogel fibers, such as a calcium phosphate cement (CPC)–hydrogel fiber structure, to improve the mechanical properties of hydrogels. Wang et al. synthesized a full-cell CPC hydrogel fiber composite material using wet spinning and mixing with a CPC paste. The mechanical properties and strength of this scaffold (8.5 ± 8.5 million Pascal) exceed that of spongy bone [45]. This is a promising result with application potential in the treatment of a wide range of bone defects in weight-bearing bones [39].

### 3.2. Hydrogel Microbeads

Traditional methods have been used for the preparation of microbeads. These technologies include emulsification, microfluidics, electrostatic droplet extrusion, coaxial air jetting, and in situ polymerization. However, traditional methods cannot provide uniform microbeads with a small size. The non-equilibrium microfluidic technique is now used for the preparation of smaller-size hydrogel beads (a size less than 100 µm) [46]. During the process of non-equilibrium microfluidics, polymer materials are inserted into a non-equilibrium water in oil (W/O) bonding that contains hydrogel molecules, in which the water molecules are dissolved into a continuous phase. The hydrogel precursors are condensed into the W/O droplets rapidly and form microbeads that are smaller than those formed under the conventional methods. Moshaverinia et al. have used injectable alginate hydrogel microbeads to encapsulate MSCs derived from dental tissue (gingival mesenchymal stem cells (GMSCs) and periodontal ligament stem cells (PDLSCs)). Mineralization of the inside of, and around, the microbeads was achieved and the cells remained viable post-implantation [47]. Furthermore, chitosan and collagen microbeads were used to encapsulate adult bone marrow stem cells via double cross-linking mechanisms by Wang and co-workers. The researchers showed that the expression of the transcription factor osterix (osx) and osteocalcin was increased and a significant deposition of bone minerals was achieved within the osteogenic medium [48].

### 3.3. Hydrogel Nanoparticles

Hydrogel nanoparticles (nanogels) are formed by physical or chemical cross-linking. They are a group of spherical nanoparticles capable of swelling in aqueous media. Nanogels have a great advantage in bone regeneration due to their good biocompatibility and desirable mechanical properties. Nanogels are typically synthesized by emulsion polymerization, such as reverse emulsion and distillation–precipitation polymerization, by rapidly stirring a solution at high temperature to disperse it steadily. In terms of their design and ease of preparation which has a wide range of polyvalent biological compositions, and their uniformity, adjustable size, stability, and high drug loading capacity, nanohydrogels are considered to be suitable systems for drug delivery. However, drugs loaded in nanogels are released quickly due to a difficulty with controlling the cross-linking point during gel formation. To solve this problem, a nanoscale structure needs to be designed to control the drug’s release [39]. Seo et al. have produced PEG nanogels with a diameter of less than 200 nanometers that turn into gel immediately after injection into the target site [49]. Yang et al. used PEG nanogels prepared by reverse microemulsion polymerization (IPMP) to carry a QK pro-angiogenic peptide. The cross-linking density of the nanogels was controlled by changing the mole fraction of the cross-linker to balance the release kinetics of the peptide QK (amino acids 17–25 of the VEGFA protein) [50]. Miahara et al. compared the use of a cholesterol-bearing pullulan (CHP) nanogel membrane with a collagen membrane in healing a parietal bone defect of an adult Wistar rat. They showed that the method increases the bone formation more effectively such that the newly formed bone in the nanogel group and the main bone are not histologically recognizable [51]. A study has applied acrylate-modulated CHP nanogels to deliver recombinant human fibroblast growth factor 18 (FGF-18) and recombinant human BMP-2 to defective bone. It was shown that the method is able to activate bone cells and eventually regenerate bone [52]. Although nanogels are suitable options for transporting proteins and growth factors to bone and inducing bone growth, the design of adjustable hydrogels that provide a high degree of mechanical stability and permanent release is essential to creating effective therapies for bone repair [39].

### 3.4. Emulsification Freeze-Drying

Several methods have been used for the fabrication and preparation of 3D scaffolds (Table 2). Conventional techniques such as blending, freeze-drying, salt leaching, and gas foaming have limited in terms of functional porosity, the requisite pore shape, geometry, and interconnectivity of scaffolds [41]. Such methodologies do not offer the native tissue’s cellular organization and totally rely on the manual seeding of the cellular components on prefabricated scaffold structures. Moreover, they are not cost-effective and their utilization is time-consuming and operator-dependent. Hence, different technologies, such as electrospinning and 3D (bio) printing, have been adopted for the fabrication of scaffolds with desired properties [53].

### 3.5. Electrospinning

Electrospinning is considered to be a very common scaffold fabrication method. This technology enables us to create nanofibrous scaffolds with interconnected pores. In electrospinning, an external electric field is applied to draw charged threads of polymer melts or solutions as very thin stream jets from a capillary tube towards a collector plate. Fibers in the nanometer diameter range are produced and deposited continuously to create a scaffold. This process has the potential to incorporate various biomolecules and composite materials [55,56]. In electrospinning, different polymers of natural or synthetic origin can be used. The natural polymers used include collagen, gelatin, silk fibroin, chitosan, silk fibroin, and hyaluronic acid. The synthetic polymers that are often used in electrospinning include polyethylene oxide, poly (lactic acid) (PLA), poly (ɛ-caprolactone) (PCL), and copolymers, such as poly (lactic-*co*-glycolic acid) (PLGA) and poly (L-lactide-*co*-caprolactone). For all of these polymers, an electrospinning methodology has been used to produce scaffolds with desired properties for drug-delivery applications and specific tissue regeneration projects [57]. Several criteria should be considered when designing electrospun bone scaffolds. These include a suitable mechanical strength and tunable biodegradation kinetics, scaffold biocompatibility and biodegradability, the pore size, inter-related open porosity for growth factors, and most importantly a sterile environment for cell seeding and eventual cell growth [13]. Coaxial electrospinning is a novel technique for the preparation of core–shell nanofibers of a gelatin–chitosan generation. The resulting scaffold has a cationic nature on the surface (chitosan), while the gelatin in its interior highly favors the attachment of cells and their proliferation. As reported by Chen et al., deposition of HA onto the surface of the nanofibers results in increased mineralization efficiency and cell adhesion [58]. Bochicchio et al. used an electrospun composite scaffold composed of poly (D, L-lactic acid) (PDLLA) and gelatin with RKKP glass and ceramics embedded inside the nanofibers. Bioactive glasses like RKKP can stimulate the formation of a hydroxycarbonate apatite layer on the scaffold to improve their interaction with the bone surface in physiological conditions and also enhance osteoconductive properties [59].

#### Surface Modification of Nanofibrous Scaffolds

Numerous surface modification approaches are utilized to achieve the desired surface characteristics of electrospun nanofibrous scaffolds. Use of these strategies can improve hydrophilicity and cellular attachment, especially for synthetic polymers [60]. The most common approaches to surface modification are plasma treatment, coelectrospinning, and surface graft polymerization [13]. Kao et al. applied a dopamine solution at pH 8.5 for coating a 3D-printed PLA scaffold. This treatment resulted in the proliferation and adhesion of human-adipose-derived stem cells (hADSCs) [61]. Depositing HA or calcium phosphate coatings onto the orthopedic implants by plasma spraying has been used for enhancing bone ingrowth. Plasma spraying, however, requires high temperatures, which may consequently alter the structural and chemical features of coatings and damage the PLA during the deposition [62].

### 3.6. Three-Dimensional (3D) Printing

Three-dimensional printing has several advantages over traditional fabrication methods. The 3D printing technology provides increased precision in designing the structure, which is required to support cell growth, proliferation, and migration. The technology enables the researcher to gain control over the pore size and shape, the level of porosity, and also the interconnectivity of pores. Highly controlled conditions for scaffold design, higher structural complexity, flexibility, and patient-specific demands are achievable [37]. The three-dimensional bioprinting process leads to printed scaffold structures with the architectural information of the defective tissue. The 3D structure of the required scaffold is obtained through instrumentational imaging that includes computerized tomography (CT) and magnetic resonance imaging (MRI). In addition, for the patient-specific and anatomically identical reproduction of the tissue, specific computer programs are used [37].

**Figure 1:**
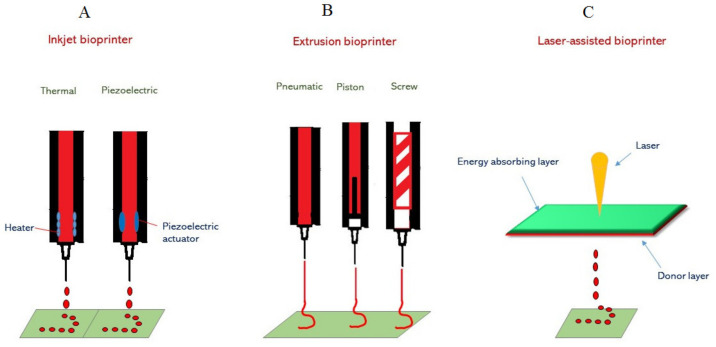
A representation of methods for 3D printing by inkjet, micro-extrusion, and laser-assisted bioprinters. (**A**) Inkjet printing (thermal and piezoelectric). In thermal inkjet printers, a heater creates air-pressure pulses resulting in the generation of droplets on the print. For piezoelectric inkjet printing, a mechanical pulse is generated by an actuator that forces the bio-ink droplets from the nozzle. (**B**) In 3D printing by micro-extrusion, three dispensing systems (pneumatic, piston-driven, and screw-driven robotics) are used to produce a continuous stream of hydrogel containing cells. (**C**) In laser-assisted bioprinting, laser energy induces bubble nucleation and forces droplets of bio-ink towards the substrate.

The major technological advancements utilized for the deposition and patterning of biological materials onto a scaffold are inkjet printing, laser-assisted printing, and micro-extrusion (Figure 1). Micro-extrusion technology is commonly used and consists of temperature-controlled material handling. It is important, however, to note that in micro-extrusion the cell viability is lower than in inkjet-based bioprinting. This is due to the fact that the cell survival rates are reduced with increasing extrusion pressure and at higher nozzle gauge values [63].

Bio-inks play a crucial role in the advancement of the technologies of functional organs and tissues in tissue engineering via 3D bioprinting. Bio-ink-related biomaterials contain collagen, agarose, alginate, gelatin-methacrylates, HA, chitin, silk, cellulose, and combinations of these materials. Nevertheless, there are major challenges to the use of bio-inks. These challenges include cell encapsulation, minimal conditions for bioprintability and cytotoxicity, high morphologic stability after printing, and biocompatibility. The designed scaffold must be able to keep its shape under wet conditions to be able to support cell proliferation and adhesion. Noh and co-workers used GelMA, HA, and hydroxyethyl acrylate (HEA) to promote the hydrogel’s mechanical stability and cellular interaction [54]. Biological factors that are involved in communication between hydrogels and stem cells include stem cell survival, polymer types, stiffness, porosity, degradation, and compatibility [64].

Strategies for bone tissue engineering provide novel approaches to bone repair through recapitulation of the developmental process of endochondral ossification. Based on previous studies, a 3D-printing modality could be exploited to engineer cartilage tissues with complex geometries and precisely controlled internal architectures to support vascularization during endochondral bone repair. Pluronic ink as a thermo-responsive hydrogel was used by Daly and coworkers to devise networks of interconnected microchannels inside MSC-laden GelMA hydrogels and printed a range of different microchannel diameters in arbitrary complex geometries [65].

Almost all scaffold fabrication methodologies lack the ability to provide the heterogeneous multiphasic porous architecture of the target tissues. Although it is possible with electrospinning technology to produce a dense network of micro/nanofibers with pore sizes and fiber diameters that closely resemble the architecture of native ECM, this technology lacks the potential to generate the expected three-dimensional structures of relevant functional tissue. On the other hand, 3D bioprinting provides scaffolds with higher dimensional control and better reproducibility. However, using this technique results in scaffolds with thicker fibers and larger pore sizes compared with electrospinning [66]. Mellor et al. used a combination of electrospinning and 3D bioprinting techniques. Using a bio-ink consisting of human-adipose-derived stem cells (hASC), they developed a reproducible and simple scaffold that incorporated both a micro-scale and a nano-scale fibrous architecture mimicking the heterogenous tissue structures. They showed that hASCs were adherent to and proliferated in the cultures, both in 3D-bioplotted and electrospun scaffolds. They showed that there was a minimal number of dead cells after 21 days in the tissue culture and that this combined scaffold structure could be easily implanted into an osteochondral defect without any problems (breaking or delamination of the scaffold) [67]. Therefore, the incorporation of electrospun nanofiber segments into bioactive materials with 3D-printed scaffolds improved cell adhesion and proliferation [68].

## 4. The Role of Exosomes in Bone Regeneration

**Figure 2:**
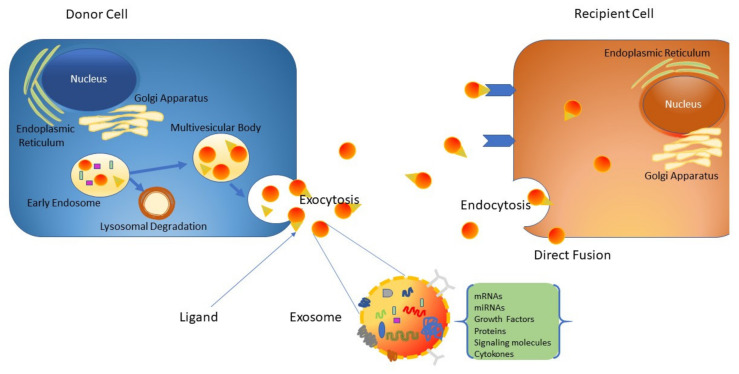
Schematic representation of exosome generation, secretion, and cargo transfer from the donor cells to the recipient cells.

The process of paracrine signaling is very important to the onset and presentation of many disease conditions, including tissue damage repair [69]. Recently, exosomes have been under intensive investigation as vital mediators of paracrine communication [70,71,72]. Exosomes are tiny extracellular nanovesicles (40–120 nm) produced from endosomal membranes of multi-vesicular bodies. They are formed by all cell types and used as a means for the exchange of cargo and signaling between cells. Different types of stem cells have been used for bone regeneration, including periodontal ligament stem cells (PDLSCs), placental stem cells (PSCs), adipose-tissue-derived MSCs, and umbilical-cord-derived MSCs [72,73,74]. Functionalization of the commercially available collagen membrane evolution (EVO) with a secretome derived from PDLSCs has been revealed to promote osteogenesis in Wistar male rats subjected to calvarias defects [73]. Additionally, PDLSCs are suitable for human clinical use as they are easy to collect and do not require invasive procedures [75]. Exosomes are generated from late endosomes by inward budding of the multivesicular body (MVB) membrane [76]. Following the invagination of late endosomal membranes, intraluminal vesicles (ILVs) within large MVBs are formed. During the inward budding process, certain proteins and cytosolic components are enclosed into the developing ILVs [77]. Upon fusion with the plasma membrane, the produced ILVs are released (exosomes) into the extracellular space (Figure 2). Alternatively, these cellular components may be taken up by lysosomes for degradation. Exosomes play a crucial role in cellular communication by the delivery of various important molecules, including mRNAs, functional microRNAs, and proteins [72,78,79]. Exosomes from stem cells have been utilized in several regenerative applications. Zhang et al. demonstrated that a scaffold of tricalcium phosphate containing exosomes (MSC-derived) could potentially enhance osteogenesis to a much higher degree than scaffolds prepared from pure tricalcium phosphate [80]. Additionally, it has been shown that exosomes from human bone marrow-derived MSCs could profoundly increase proliferation, migration, and osteogenic differentiation through miRNA profiles in recipient stromal cells [81]. Moreover, exosomes are critically important in inflammatory reactions after injury; they boost the regeneration and repair of damaged tissue [82]. Data provided by various studies indicate that the addition of particular ligands to the surface of exosomes increases their affinity towards specific cell types and makes them susceptible to selective capture by target cells [83].

Exosomes have several advantages as nanocarriers. These advantages include their slightly negative zeta potential, which is required for a long circulation time, their external molecular similarity to cell membranes, their very small size, which renders them suitable for penetration into deep organs and tissues, and their high potential and capacity to escape degradation in circulation or clearance by the immune system [84,85].

Addition of therapeutic or medicinal agents into exosomes has been performed by two different mechanisms: active and passive encapsulation. In passive encapsulation, the target drug is incubated with exosomes, while in active modification a mechanical shear force is used to change the integrity of the exosome membranes, which permits the drug molecules to diffuse into the exosomes during the process of membrane alteration [83].

### 4.1. Role of Exosomes in MSC Differentiation

MSC-derived exosomes have been exploited for different therapeutic approaches to vascular diseases such as stroke (cerebral infarction), cardiac fibrosis, and liver ischemia/reperfusion injury [86]. It has been shown that exosomes with enriched miRNA content could be essential players in intercellular communication in the nervous system. For example, through miR-132, exosomes may become involved in cell-to-cell signaling and mediate neural regulation of brain vascular integrity [87]. It has been shown that exosomes derived from human umbilical cord MSCs (hucMSC exosomes), human adipocyte MSCs (haMSC exosomes), and human induced pluripotent stem-cell-derived MSCs (hiPS-MSC exosomes) promote skin wound healing by delivering various functional soluble signaling molecules, such as cytokines, RNAs, and proteins [88]. By exploitation of exosomes, the cancer cell secretome modulates stromal cell fate and induces the differentiation fibroblast cells into myofibroblast lineages.

### 4.2. The Role of Exosomes in Osteoblast Proliferation and Activity

**Figure 3:**
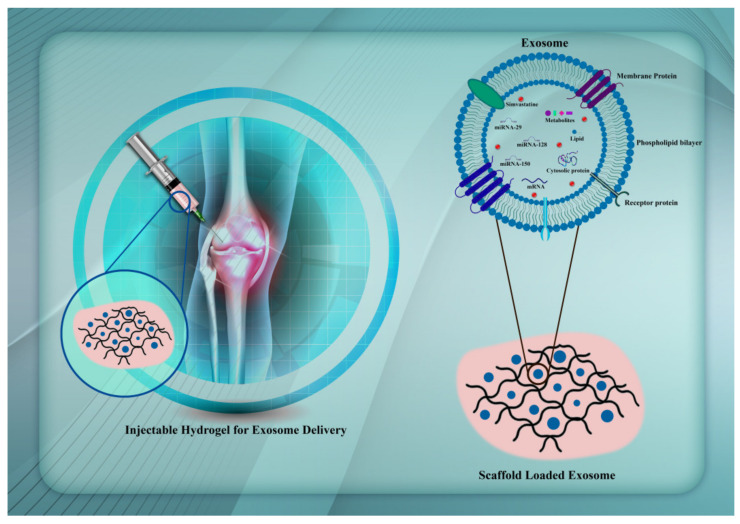
Targeting miRNAs/simvastatin in mesenchymal stem cells using exosomes to enhance osteogenesis.

Exosomes may trigger the differentiation of stem cells without the help of growth factors and cytokines. Wang and co-workers obtained exosomes from undifferentiated human mesenchymal stem cells (hMSCs) and compared their miRNA contents after osteogenic lineage differentiation [89]. The expression levels of miRNAs involved in osteogenic differentiation, such as miR-181a, miR-221, miR-885-5p, miR-155, and miR-320c, were lower in exosomes isolated from differentiated cells, while in undifferentiated cells the expression levels of miR-148a, miR-199b, miR-218, miR-135b, let-7a, miR-219, miR-203, miR-299-5p, and miR-302b were increased [89]. Many investigations have indicated that functional miRNAs may be selectively loaded into exosomes, which in turn serve as important regulators of angiogenesis and other endothelial cell functions [90]. The microRNA miR-885-5p negatively regulates the osteogenic differentiation of bone-marrow-derived MSCs by inhibiting the functions of bone morphogenetic protein 2 (BMP2). Furthermore, the suppressive effects of miR-885-5p on osteogenic differentiation involve the Wnt pathway as they result in downregulation of Wnt5a mRNA [91]. On the other hand, let-7a positively regulates osteogenesis lineage differentiation by influencing high mobility group AT-hook 2 (HMGA2) and blocking the adipogenic differentiation of MSCs [92]. In a similar way, miR-218 positively regulates osteogenic differentiation. This effect has an impact on the Wnt/ß-catenin pathway, which in turn is an important player in the osteogenesis of adipose-derived stem cells [93]. The stability and structural retention of exosomes are very important for their useful application (Figure 3).

Chitosan hydrogels have high biocompatibility, mimic the natural extracellular matrix, and facilitate cell migration, adhesion, and proliferation. Zhang et al. used thermosensitive chitosan hydrogels to improve the in vivo retention and stability of exosomes as well as enhance their therapeutic capacity [94]. In particular, 3D-printed PLA loaded with human gingival MSCs (hGMSCs) and EVs was shown by a MicroCT assessment to enhance bone regeneration and the vascularization process [95]. Table 3 summarizes the therapeutic effects of loading osteogenic miRNAs into exosomes in pre-clinical animal model systems.

## 5. Hydrogels for Exosome Delivery

The biodistribution of purified, unformulated exosomes has been studied in animal models. Different routes of administration, including intravenous (i.v.), intraperitoneal (i.p.), subcutaneous (s.c.), intranasal, and retro-orbital, were used to evaluate the disposition and exosome kinetics in vivo [94]. Exosomes have more advantages compared with stem cells for tissue repair. They remain highly stable for a long time without alteration of the biological activity. They can target organs quickly, initiate tissue repair, and preserve a variety of bioactive components from degradation [101]. Exosomes reduce the risk of iatrogenic tumor formation due to their non-self-replicating feature and reduce the embolism formation accompanied by injection of MSCs. Purified, unformulated exosomes are taken up by the reticuloendothelial system and cleared from the body in a relatively short time [102]. To overcome these limitations, biodegradable hydrogels can play a protective role and act as exosome carriers and exosome delivery reservoirs at the site of entry, resulting in a more sustained therapeutic effect. Moreover, exosomes formulated in biocompatible and biodegradable hydrogels can be introduced near or at the target tissue site and facilitate localized delivery of a high concentration of therapeutic molecules entrapped in exosomes [11]. Due to the structural and physio-chemical properties of hydrogels, it is also possible to tune the degradation rate of hydrogel matrices and control the release and functional properties of the embedded exosomes. Moreover, as biodegradable hydrogels are biologically compatible and resemble the intracellular matrix, they can be considered to be excellent candidates for exosome encapsulation for various therapeutic applications. These advanced hydrogel–exosome formulation platforms may also provide unique modalities for tissue engineering, including bone repair applications [103]. Exosomes’ therapeutic efficacy strongly depends on the design and function of the hydrogel. Zhang et al. prepared and tested an injectable thermosensitive chitosan hydrogel. They showed that a reliable hydrogel with biodegradable and biocompatible properties can improve the stability and in vivo retention of exosomes from human-placenta-derived MSCs (hPMSCs). The hydrogel also enhanced the therapeutic efficacy for angiogenesis. Studies involving various hydrogel systems for the delivery of exosomes and their potential interactions have attracted a lot of attention among tissue engineering scientists. [94]. In one study, synovium-derived MSCs encapsulated in chitosan were found to overexpress miRNA-126-3p in the presence of hydroxyapatite (HAp) nanoparticles. The miRNA-126-3p overexpression delayed the release of MSCs from the exosome (from 2 days to 6 days). Integrating the exosomes derived from BMP2-activated macrophages into titanium nanotubes has proven to be important to the enhancement of osteogenesis [104]. Yang et al. used sensitive hydrogels with a self-healing ability for the continuous release of human umbilical cord mesenchymal stem cell (hUCMSC)-derived exosomes. Their results reveal that exosomes loaded in an injectable hydrogel alginate (ALG) and hyaluronic acid (HA) (HA-ALG) can improve osteogenesis in a rat model of a calvarial bone defect. The advantages of this hydrogel include the ability to self-repair, excellent surface morphology, high biocompatibility, low toxicity, a suitable scaffold for exosomes, and a good ability to repair bone. Additionally, the combination of a hydrogel and an exosome together can promote the healing of damaged bones, BMP2 deposition, deposition and maturation of bone collagen, and increased angiogenesis in the SD rat model [105]. Table 4 presents some examples of hydrogel–exosome delivery systems used in tissue engineering studies.

## 6. Future Perspectives

Future studies will support smart biomaterials and improve our understanding of the interaction between biomaterials and the cellular response, which will enable them to adjust to environment changes. Furthermore, the biocompatible hydrogel CRISP system based on endonuclease cas12a containing single-stranded DNA in a polyethylene glycol hydrogel could improve the sustained release of drugs, nanoparticles, and cells [110]. Moreover, intelligent biomaterials could reduce the transplant rejection rate via the release of anti-inflammatory cytokines. For example, the incorporation of strontium into bio-glass can modulate the immunoresponse [111]. Taken together, further investigations that consider the sustained release of nanoparticles and RNAs from a hydrogel to decrease inflammation and improve mechanical properties will be beneficial for bone regeneration.

## 7. Conclusions

Bone tissue engineering has considerably and practically advanced in the last few years. In this context, hydrogels of a natural, synthetic, or hybrid origin are attractive candidates to be incorporated in therapeutic applications of bone tissue regeneration. Their biocompatibility, biodegradability, and mechanical properties must be evaluated and their interaction with the surrounding tissue is also of importance. Additionally, incorporation of exosomes into hydrogels could significantly stabilize and retain exosomes at the injury sites and have an impact on the maintenance of the stability of exosomal contents of proteins and miRNAs under physiological conditions. Nevertheless, for the realization of the potential of bone tissue repair, many technical challenges remain. These challenges include the scalable manufacturing of biomimetic scaffolds, efficient methods for cellular growth and differentiation, and efficient delivery of biologically active molecules.

## Figures and Tables

**Table 1:** A representative list of the most common commercially available natural hydrogels for bone regeneration.

Natural of Hydrogel	Advantages	Disadvantages
Hyaluronic acid	Advanced tissue repair, angiogenesis, biocompatible for 3D printing	High degradation rate in vivo
Alginate	Biodegradable	Poor mechanical properties
Chitosan	Biocompatible, low toxicity, suitable mechanical properties	Lack of thermal stability
Collagen	Biodegradable, biocompatible	Low mechanical strength
Gelatin	Low immunogenicity, high water solubility, high degree of cell attachment	Low stability, poor mechanical properties, lack of thermal stability
Pectin	Promotes the nucleation mineral phase if immersed in biological fluids	Difficult to standardize in an economic way
Dextran	High biocompatibility, good adhesion of vascular endothelial cells	Inability to provide a surface to support cell adhesion and growth

**Table 2:** Benefits and disadvantages of fabrication methods.

Scaffold Fabrication	Advantage(s)	Disadvantage(s)	Ref.
Electrospinning	Simple method Uniform and aligned fibers 80–95% porosity Capability to produce fibers with a diameter of 100 nm to several microns <80% cell viability Relatively inexpensive technique	Difficult to produce high voltage Toxic solvents Packaging, shipping, handling	[13]
Freeze drying	Simple and cost-effective 30–80% porosity with a diameter of 50–40 nm High cell viability	Cannot provide regular porosity Have a long processing time	[13]
3D Printing	Fabricates the desired structure	Needs a 3D printer Toxic solvents Lack of mechanical strength	[54]
Hydrogel fibers	High surface-to-volume ratio Rapid response and immobility Great potential in bone regeneration	Poor mechanical strength High swelling ratio and rapid drug release	[39]
Hydrogel microbeads	High capability for encapsulating stem cells and drugs	Low osteoconductivity/osteoinductivity	[39]
Hydrogel nanoparticles	Good biocompatibility Desirable mechanical properties Easy to design and prepare Surface with a wide range of polyvalent biological compositions High drug loading capacity	The possibility of drug leakage Poor mechanical strength	[39]

**Table 3:** Summary of recent studies on loading osteogenic miRNAs into exosomes in vivo.

Donor Cell	Recipient Model	Rout	Dose Exosome	miRNA/mRNA	Target Gene	Effect	Ref.
BMSCs	C57BL/6J mice	Intravenous injection	100 µg protein	miR-29a	VASH1, COL1A1, VEGFA, RUNX1T1	Increase osteogenesis Increase angiogenesis	[96]
BMSCs	Sprague–Dawley (SD) rats	Intravenous injection	200 µg protein	miR-128-3p	Runx2	Increase osteogenesis	[97]
BMSCs	Sprague–Dawley (SD) rats	Intravenous injection	100 µg protein	miR-150-3p	Runx2, Osterix, ALP and osteocalcin	Increase osteogenic differentiation	[98]
BMSCs	Balb/c mice	Intravenous injection	200 µg protein	antagomir-188	RUNX2, osterix (Sp7), osteocalcin (Bglap)	Increase osteogenic differentiation, Decrease adipogenic differentiation	[99]
BMSCs	Sprague–Dawley (SD) rats	Intravenous injection	100 µg protein	miR-935	STAT1	Increase osteogenic differentiation	[100]
GMSCs	Wistar rats	3D printing	0.5 µg/µL	miR-2861,210	VEGFA, RUNX2 COL1A1	Increase osteogenesis Increase angiogenesis	[95]

**Table 4:** A representative list of different biomaterial characteristics used for exosome formulation and delivery.

Type	Retention Rate (%)	Release Time	Cross Link	Loading Molecules	Feature	Ref.
Thermosensitive chitosan	98	12 h		Encapsulating exosomes	Increase in cell adhesion, migration, and proliferation, a good carrier for sustained-release exosomes	[103,106]
Hydroxyapatite Alginate (HA-ALG) hydrogel		14 days	Schiff-base reaction	Encapsulating exosomes	Increase in osteogenic and angiogenic abilities	[105]
Titanium nanotubes				Encapsulating exosomes	Increase in osteogenic abilities	[107]
Hyaluronic acid (HA)	90	14 days	Photoinduced imine cross-linking	Encapsulating exosomes	High water content, swelling behavior, and biocompatibility, modulated 3D networks and high cartilage matrix mimetics, significantly facilitates the migration of cells to and promotes cell deposition at cartilage defect sites	[108]
Hydrogel (2% thermosensitive chitosan)	86	2 days		Encapsulating exosomes	Improvement in in vivo retention and stability of exosomes	[109]
Tricalcium phosphate		5 days			Osteo-inductive biomaterial and a biodegradable ceramic	[80]
Hydroxyapatite (HAp) nanoparticles in chitosan	Sustained release	6 days		Encapsulating exosomes	Angiogenesis antibacterial activity	[104]

## Data Availability

Data are contained within the article.
